# Establishment and Characterization of a Novel Fibroblastic Cell Line (SCI13D) Derived from the Broncho-Alveolar Lavage of a Patient with Fibrotic Hypersensitivity Pneumonitis

**DOI:** 10.3390/biomedicines9091193

**Published:** 2021-09-10

**Authors:** Paolo Giannoni, Marco Grosso, Giuseppina Fugazza, Mario Nizzari, Maria Cristina Capra, Rita Bianchi, Roberto Fiocca, Sandra Salvi, Fabrizio Montecucco, Maria Bertolotto, Franco Fais, Mario Salio, Emanuela Barisione, Daniela de Totero

**Affiliations:** 1 Department Experimental Medicine, University of Genoa, 16132 Genoa, Italy; paolo.giannoni@unige.it (P.G.); franco.fais@unige.it (F.F.); 2 Interventional Pulmonary Unit, San Martino Policlinico Hospital, IRCCS for Oncology and Neurosciences, 16132 Genoa, Italy; marco.grosso@hsanmartino.it (M.G.); emanuela.barisione@hsanmartion.it (E.B.); 3 Department Internal Medicine and Medical Specialties, University of Genoa, 16132 Genoa, Italy; Giuseppina.Fugazza@unige.it (G.F.); fabrizio.montecucco@hsanmartino.it (F.M.); maria.bianca.bertolotto@unige.it (M.B.); 4 Department Internal Medicine and Centre of Excellence for Biomedical Research, University of Genoa, 16132 Genoa, Italy; mario.nizzari@unige.it; 5 Molecular Pathology Unit, San Martino Policlinico Hospital, IRCCS for Oncology and Neurosciences, 16132 Genoa, Italy; mariacristina.capra@hsanmartino.it; 6 Anatomic Pathology Unit, San Martino Policlinico Hospital, IRCCS for Oncology and Neurosciences, 16132 Genoa, Italy; bianchirita22@gmail.com (R.B.); roberto.fiocca@hsanmartino.it (R.F.); sandra.salvi@hsanmartino.it (S.S.); 7 Department of Surgical and Integrated Diagnostic Sciences, University of Genoa, 16132 Genoa, Italy; 8 Italian Cardiovascular Network, San Martino Policlinico Hospital, IRCCS for Oncology and Neurosciences, 16132 Genoa, Italy; 9 S.C. Malattie Apparato Respiratorio, Azienda Ospedaliera Nazionale Antonio, Biagio e Cesare Arrigo, 15121 Alessandria, Italy; mario.salio@ospedale.al.it

**Keywords:** hypersensitivity pneumonitis, fibrosis, myofibroblasts, pirfenidone

## Abstract

Hypersensitivity pneumonitis (HP) is a diffuse interstitial lung disease (ILD) caused by the inhalation of a variety of antigens in susceptible individuals. Patients with fibrotic HP (fHP) may show histopathological and radiological manifestations similar to patients with idiopathic pulmonary fibrosis (usual interstitial pneumonia-like pattern of fibrosis) that are associated with a worse prognosis. We describe here the establishment and characterization of a fibroblastic cell line derived from the broncho-alveolar lavage (BAL) of a patient with fHP, a 53 year old man who presented at our Pneumology Unit with cough and dyspnea. The fHP diagnosis was based on international criteria and multidisciplinary discussion. Primary fibroblasts were expanded in vitro until passage 36. These fibroblasts displayed morpho/phenotypical features of myofibroblasts, showing high positivity for α-smooth muscle actin, type I collagen, and fibronectin as determined by quantitative RT-PCR and cyto-fluorographic analysis. Cytogenetic analyses further evidenced trisomy of chromosome 10, which interestingly harbors the FGF2R gene. To our knowledge, this is the first fibroblastic cell line derived from an fHP patient and might, therefore, represent a suitable tool to model the disease in vitro. We preliminarily assessed here the activity of pirfenidone, further demonstrating a consistent inhibition of cells growth by this antifibrotic drug.

## 1. Introduction

Progressive fibrosing interstitial lung diseases (ILDs) are associated with high mortality; in particular, patients developing the fibrotic form of hypersensitivity pneumonitis have a poor prognosis. Hypersensitivity pneumonitis is difficult to diagnose since clinical symptoms and pathologic or radiologic imaging often resemble those of other ILDs [1,2,3]. Hypersensitivity pneumonitis is caused by an exaggerated immune reaction developing in a small percentage of individuals that are sensitized by a large variety of environmental antigens [4]. More than 300 are the molecules specifically recognized as causing HP, but others, putatively involved in the disease, are still unknown. Among the known antigens that frequently cause HP, there are microbial agents (e.g., *Saccaropolysphora rectivirgula*), fungi (e.g., *Trichosporum cutaneum*) [5], animal proteins (mostly avian) [6], or chemical molecules (e.g., di-isocyantes) [7]. HP may be classified as acute or chronic. Symptoms of HP in its acute form occur 4–6 h after exposure to a large quantity of allergen. However, acute HP often resolves in some hours, with a maximum of a few days, if the exposure to the antigen is ceased. On the contrary, fibrotic HP develops in response to a long-term exposure to low levels of antigen; it usually occurs with more subtle symptoms, gradually leading to a rapid decline in lung function and to early mortality [8]. In fHP, fibroblasts progressively expand and infiltrate the lung interstitium, finally leading to loss of the vital lung capacity. Fibroblasts, chronically infiltrating the lung in fHP, assume typical features of myofibroblasts, thus overexpressing alpha-smooth muscle actin (α-SMA) and producing high levels of collagen. These markers are also typically expressed in cancer-associated fibroblasts (CAFs); notably, the overexpansion of CAFs in the tumor microenvironment of the lung is strongly associated with poor prognosis [9]. Hypothetically, fibrosis of lung tissue might predispose fHP patients to cancer, as already demonstrated in IPF. Kuramochi et al., in a group of 104 fHP patients, identified 11 individuals (15 lesions) with lung cancer (10.6%). Interestingly the most prevalent histopathological type of lung cancer was squamous cell carcinoma [10], which is also dominant in IPF-associated lung cancer [11,12].

Standard treatments for HP currently consist of withdrawal of the offending antigen and systemic corticosteroid therapy. In fHP, however, an anomalous fibrotic process strongly affects the vital function of the lung, progressively leading to the destruction of the alveolar architecture. These observations have, therefore, recently stimulated the evaluation of antifibrotic therapies which may ameliorate the pulmonary functions.

In this study, we established a cell line (SCI13D) derived from the broncho-alveolar lavage (BAL) of a patient with a diagnosis of fHP. The cell line was expanded in vitro until passage 36 and was phenotypically, morphologically, and functionally characterized. As far as we know, this is the first cell line developed from an fHP patient and, therefore, it could be of help in modeling novel therapies for this disease. In this prospect, we attempted to downregulate the expression of myofibroblasts markers and inhibit the proliferation and migration of SCI13D using pirfenidone.

## 2. Materials and Methods

### 2.1. Patient, Bronchoalveolar Fluid Collection, and Histological Sample Assessment

The present study was approved by the Regional Ethics Committee (ILDFIBRO020) and conducted according to the current national and international guidelines; within the study, biological samples were anonymized prior to processing. The diagnosis of fHP for the patient was based on international criteria. After multidisciplinary discussion of the Interstitiopathy Lung Disease (ILD) group of the case, and after subscription of an informed consent form, explicitly authorizing the use of biological samples and any derivative thereof for research purposes, the patient underwent a transbronchial cryobiopsy [13] with bronchoalveolar collection (BAL) for diagnostic purposes (cyto-fluorographic and histological analysis). Aliquots of the BAL collection, unused for diagnosis, were further processed for additional research procedures.

For histology, all the four samples obtained with cryoprobe were fixed in formalin, routinely processed, embedded in paraffin, and cut using a microtome, obtaining three levels of sections, before staining with hematoxylin and eosin (H&E). Adjunctive histochemical staining (Alcian blue PAS and Mallory’s trichrome) and immunohistochemistry (Cytokeratin 7, all provided by Ventana Medical System, Inc., Oro Valley, AZ, USA) were performed to facilitate the diagnostic process. BAL cytology was also examined for diagnostic purposes (*data not shown)*.

### 2.2. Cell Line Establishment and Culture

Cells were first pelleted from the BAL fluid and then seeded in a 24-well plate in complete medium (RPMI-1640 + 10% FCS; Lonza, Walkersville, MD, USA) to be cultured at 37 °C in 5% CO_2_. Fibroblast growth factor 2 (FGF2; 3 ng/mL; Miltenyi Biotech GMbH Friederich-Ebert, Germany) was added 1 day after. The medium was changed once a week, and FGF2 was also added. After 3 weeks, a consistent number of spindle-shaped cells appeared, growing attached to the bottom of a well to form a colony. Cells were, therefore, expanded and grown until confluence. After each trypsin treatment (Euroclone S.p.A., Milan, Italy) detached fibroblasts were washed, collected, and further expanded in 24- or six-well plates. Aliquots of these cells were then assessed for the expression of markers specific for fibroblast/myofibroblast differentiation by quantitative RT-PCR or through staining with specific moAbs and subsequent cyto-fluorographic analysis, as described below. Fibroblasts were in part frozen or continuously expanded until passage 36.

### 2.3. mRNA Extraction and Quantitative Real-Time RT-PCR

Cells were washed in phosphate-buffered solution (PBS; Euroclone), detached by trypsinization, and collected by centrifugation (400× *g*). Total mRNA was extracted by using the GeneUP^TM^ Total RNA Kit (BiotechRabbit GmbH, Berlin, Germany), following the manufacturer’s instructions. Upon generation of the cDNA pool for each sample, by using the SuperScript^TM^ III First-strand synthesis system for RT-PCR Kit (Invitrogen; Milan, Italy), real-time quantitative RT-PCR analysis was subsequently undertaken to evaluate the relative expression of target genes; a SYBR-Green RealMasterMix SYBR ROX 2.5× (5-Prime GmbH, Hamburg, Germany) was used in an Eppendorf Mastecycler Realplex^2^ apparatus. Reactions were performed in triplicate for each treatment/sample, according to the following settings: a single denaturation step at 95 °C for 3 min, followed by 45 cycles at 94 °C for 30 s, 60 °C for 30 s, 72 °C for 40 s, and a final step at 72 °C for 7 min. The specificity of each reaction was assessed by the melting curve analysis. The expression of each gene was normalized to the endogenous housekeeping gene glyceraldehyde-3-phosphate dehydrogenase (GAPDH). Primer sets for target genes were derived from previously published sequences, such as for type 1 collagen [14], or purposely designed, such as for the alpha-smooth muscle actin (α-SMA; forward primer: TGGAAAAGATCTGGCACCAC, reverse primer: CTCAAACATAATTTGAGTCAT), fibronectin (FN; forward primer: TACACTGGGAACACTTACCG; reverse primer: CCAATCTTGTAGGACTGACC), fibroblast growth factor-2 receptor (FGF-2R; forward primer: AGACAGGTAACAGTTTCGGCT, reverse primer: CAGTGTCAGCTTATCTCTTGG), CDK1N1A (P21; forward primer: TGAGCCGCGACTGTGATG, reverse primer: GTCTCGGTGACAAAGTCGAAGTTC), and CDKN2A (P16; forward primer: GTGGACCTGGCTGAGGAG, reverse primer: CTTTCAATCGGGGATGTCTG). The expression level of each target gene among different samples and/or culture conditions was normalized by the level of the corresponding gene in control cultures.

### 2.4. Morphology, Cyto-Fluorographic, and Immunofluorescence Analyses

Morphological characteristics of live cultured cells, expanded in 24- or in six-well plates, were observed under an inverted Olympus CKX-41 microscope ad acquired using a Nikon Digital Sight DS-5Mc camera equipped with the NIS-Elements F2.20 software. Images of the cell gross morphology were acquired after fixing cells with 4% paraformaldehyde in PBS for 15 min and staining them with a Methylene Blue solution (1%, in borate buffer, pH 8.8) for an additional 15 min at RT. Phenotypical analysis was performed by staining detached cells with specific monoclonal or polyclonal antibodies (moAbs) such as FITC-conjugated anti-human CD105 (ImmunoTools GmbH, Friesoythe, Germany) or -vimentin (M7020 DakoCytomation, Glostrup, Denmark), -fibronectin (DP3060, ACRIS, LiStarfish, Milan, Italy), -procollagen-1 (SP1.D8; Developmental Studies Hybrydoma Bank, Univesity of Iowa; Iowa City, IA, USA), -type III collagen (MAB3392; Millipore Australia Pty, Ltd., Boronia, Victoria 3155, Australia), -α-smooth muscle actin (clone 1A4; Invitrogen-Thermo Fisher Scientific, Milan, Italy), -p21 Waf1/Cip1(sc-187, Santa Cruz Biotechnology Inc., Dallas, TX, USA) or -α-smooth muscle actin directly conjugated to Alexa (ab5694; Abcam, Cambridge, UK; Prodotti Gianni S.p.A., Milano, Italy) or FITC-conjugated anti-Ki-67 (BD Pharmingen Inc., San Diego, CA, USA) For cyto-fluorographic assessment, cells were first stained for the CD105 membrane antigen. After 30 min of incubation at 4 °C, the cells were washed with PBS + 2% FCS, fixed with 1% PFA, and incubated again on ice in the dark for 15 min. After two washes with PBS + 2% FCS, the cells were stained with the Alexa466-conjugated α-SMA antibody or with anti-human vimentin, fibronectin, collagen-1, or collagen-3 antibodies. Cells were, therefore, incubated on ice for 30 min more. After this incubation, the cells were washed and, then, when necessary, stained with a specific anti-mouse or anti-rabbit secondary PE-conjugated antibody (Southern Biotechnology, Birmingham, AL 35226, USA) for 30 min. Cyto-fluorographic analysis was then performed using an FACSCanto cytofluorimeter (Becton Dickinson; Franklin Lakes, NJ, USA).

Immunofluorescence was also performed on fibroblasts (SCI13D and HF) previously grown in chamber slides (Thermo Fischer Scientific; Rodano (MI), Italy). The cells were first fixed with 4% PFA in PBS for 15 min. After three washes with PBS + 2% FCS, cells were incubated for 30 min in the dark with an anti-human Actin moAb (DM001P, Acris Antibodies; Li.StarFISH, Carugate (MI), Italy). After three more washes, fibroblasts were stained with an isotype-specific secondary antibody fluorescein conjugate (Southern Biotechnology). Cell nuclei were identified by standard staining with 4′,6-diamidine-2′-phenylindole dihydrochloride (DAPI; Merck Serono S.p.A., Rome, Italy).

### 2.5. Immunocytochemistry of the SCI13D Cell Line

Cytological analyses of SCI13D were performed with standard hematoxylin and eosin staining or with immunocytochemical probing for vimentin, smooth muscle actin, and cytokeratin AE1–AE3 (Ventana Medical Systems, Inc., Oro Valley, AZ, USA).

### 2.6. Proliferation and Growth Kinetic Determination

Cells were seeded in a six-well plate at a density of 3 × 10^4^/well, expanded, harvested, and counted after 14 days using 0.1% trypan blue and a Neuerbauer count chamber. Fibroblasts were then reseeded in 24-well plates to a density of 10^4^ cells/mL in complete medium and left to adhere for 24 h. Non-adhered cells were washed away, and treatments were performed by exposing cells to complete medium supplemented with pirfenidone (150–450 μg/mL; D.B.A. Italia, Segrate, Milan, Italy), TGF-β1 (5 ng/mL; Miltenyi Biotech GMbH Friederich-Ebert, Germany), or both. At each assessed timepoint, cells were thoroughly washed with sterile PBS and incubated for 4 h at 37 °C with complete culture medium supplemented with 10% Alamar Blue^TM^ (Invitrogen; Milan, Italy). Subsequently, aliquots of the supernatants were drawn and assessed spectrophotometrically at 570 and 600 nm in a Spectra MR Dynex apparatus, while cells were washed and replenished with complete medium. Determinations were performed in duplicate for each well and treatment, for each timepoint (0, 1, 4, and 7 days). In selected experiments, wherever indicated, FGF2 was added at different concentrations (3 or 10 ng/mL).

### 2.7. Wound Sratch Test

Fibroblasts were seeded in 24-well plates to a density of 10^4^ cells/mL, and then left to adhere and proliferate to semi-confluency in complete/treatment medium. A scratch was then made onto the cell layer, throughout the full length of the diameter of each culture well, by using a sterile 200 μL micropipette tip. Pirfenidone treatment was contemporarily started (150, 300 or 450 μg/mL), wherever needed. At any assessed timepoint, the number of cells encompassed by the lesion area was used as a parameter to define the fastest and most efficient growth conditions among the experimental settings assessed. Images of cells filling the gaps were acquired 0, 6, 24, 36, and 48 h after generating the lesion. The original boundaries of each lesion area were superimposed to frames captured from the same plate in the same position at each experimental timepoint; cells within boundaries were then counted using the National Institute of Health ImageJ free-software 1.48v (http://imagej.nih.gov/ij access on 1 September 2021).

### 2.8. Determination of the Total Cellular F-Actin with/without Pirfenidone Treatment

SCI13D fibroblasts were cultured in eight-well chamber slides (Nunc) in a total volume of 300 μL of culture medium. The cells were then treated with TGF-β 5 ng/mL, pirfenidone 300 μg/mL, or TGF-β + pirfenidone for 72 h. Cells were, therefore, fixed in 2.5% PAF, permeabilized with Triton × 100, and stained with Alexa-Fluor 555-conjuged phalloidin (Thermo Fischer Scientific; Rodano (MI), Italy).

The content of F-actin was determined by fluorescence microscopy (Nikon Optiphot-2; Nikon, Melville, NY, USA), as described previously [15]. Image capturing was performed with a Hamamatsu color-chilled 3 CCD camera. Identical camera settings (time of exposure, brightness, contrast, and sharpness) and an appropriated white balance set according to the fluorescence filter were used. Pictures were acquired and analyzed using Image-Pro Plus 4.0 (Media Cybernetics Inc., Rockville, MD, USA). Nuclei were counterstained with DAPI. The mean fluorescence density was determined from a linear measurement of cell fluorescence in randomly chosen fields of each slide (six fields per slide). Results depict the mean ± SEM of the fluorescence densities (the relative intensity was normalized counting cells nuclei of each field for each individual slide).

### 2.9. β-Galactosidase Assessment

Senescence-associated β-galactosidase is a manifestation of residual lysosomal activity at a suboptimal pH (pH6), which becomes detectable due to the increased lysosomal content in senescent cells. The senescence-associated β-galactosidase assay is, therefore, widely used as a biomarker of senescent cells [16]. SCI13D fibroblasts cultured in six-well plates were first washed with PBS 1× and then fixed with PFA 4% for 5 min at room temperature. Cells were rinsed with PBS and incubated overnight in the incubator at 37 °C in SA-β-Gal staining solution containing 1 mg·mL^−1^ X-gal, 40 mM citric acid/sodium phosphate buffer pH 6.0, 5 mM potassium ferricyanide, 5 mM potassium ferrocyanide, 150 mM sodium chloride, and 2 mM magnesium chloride in water. Cells were then rinsed with PBS and observed at an inverted bright field microscope. At least five images for each well were acquired as described above.

### 2.10. Cytogenetics

The patients’ fibroblasts at the third passage were detached from the plate with trypsin and collected. PHA-activated lymphocytes from peripheral blood obtained from the same subject were also analyzed. A chromosome study was performed on the SCI13D cell line and on peripheral blood using standard cytogenetic techniques. Briefly, cells were exposed to colcemid (0.04 μg/mL) for 30 min at 37 °C and to hypotonic treatment (0.075 M KCl) for 15 min at room temperature. Cells were fixed in a methanol and acetic acid (3:1 volume/volume) mixture for 15 min and then washed three times in the fixative. The slides were air-dried, and karyotyping was carried out on QFQ-banded chromosomes and was reported using the ISCN 2020 nomenclature. Fluorescence in situ hybridization (FISH) was performed using a *human chromosome 10* alpha satellite probe (ZytoLight CEN10 probe, ZytoVision GmbH, Bio-Optica, Milano, Italy) according to the manufacturer’s recommendations, and the automated VP2000 processor was utilized for paraffin-embedded tissue studies, allowing high performance of all the procedures [17].

### 2.11. Short Tandem Repeat Analysis

Short tandem repeat (STR) profiling was performed to verify the authenticity, as well as the unicity, of the cells using the services provided by Banca Cellule Interlab Cell Line Collection (ICLC; IRCCS Ospedale Policlinico San Martino, Genoa, Italy). The STR profiles of the cell line and of the PBMCs of the patient were determined. Fifteen highly polymorphic STR loci plus amelogenin (Cell ID^TM^System, Promega Italia, 20126 Milano, Italy) were used. Detection of amplified fragments was obtained using the ABI PRISM 3100 Genetic Analyzer. Data analysis was performed using Gene Mapper software, version 3.2 (Applied Biosystems, Whaltman, MA, USA).

### 2.12. Statistical Analysis

Whenever needed, Student’s *t*-test was used to determine the significance of *p*-values as follows: 0.05 ≥ *p* > 0.01 (*); 0.01 ≥ *p* > 0.001 (**); *p* ≤ 0.001 (***).

## 3. Results

### 3.1. Patient’s Clinical and Diagnostic Features

The patient (53 years old; male) presented at the Pneumology Unit of our Institute with cough and moderate dyspnea. The CT scan showed an alternative diagnosis pattern according to the Official ATS/ERS/JRS/ALAT clinical practice guidelines [18], with predominant ground glass opacity and peribronchovascular distribution. The functional test showed a moderately restrictive form. Transbronchial cryobiopsy, in the lower right lobe, and BAL collection, in the middle lobe, were performed for diagnostic purposes after multidisciplinary discussion. Cyto-fluorographic analysis evidenced the presence of lymphocytosis (26%). Diagnosis of fHP, first based on lymphocytosis detection in BAL and on a UIP pattern with abundant inflammation, was also confirmed by histological sample assessment, as described below.

**Figure 1:**
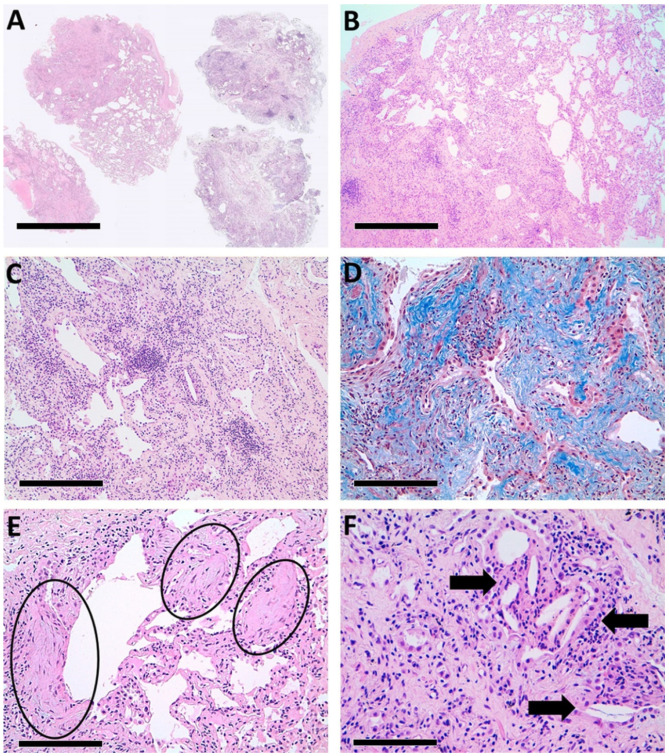
Most relevant histological lesions found in the patient’s biopsies. (**A**) The low-power image shows that the lesions affect all four cryobiopsies (H&E, ×2; scale bar = 150 µm); (**B**) the parenchymal involvement is irregular (H&E, ×4; scale bar = 60 µm); (**C**) diffuse interstitial fibrosis is accompanied by a partly nodular inflammatory infiltrate (H&E, ×10; scale bar = 25 µm); (**D**) Mallory’s trichrome stain highlights (in blue) collagen fibrosis (×20; scale bar = 12.5 µm); (**E**) the fibroblastic foci (circled) are numerous (H&E, ×20; scale bar = 12.5 µm); (**F**) some cholesterin clefts (arrows) are surrounded by giant cells (H&E, ×40; scale bar = 6.25 µm).

The putative fHP diagnosis was supported by histological examination of the cryobiopsy-derived samples. Four samples were examined and showed a spatially heterogeneous fibrotic pattern (Figure 1A,B) with sparse fibroblastic foci and intervening normal lung parenchyma. In this context, diffuse inflammatory infiltration, composed of lymphocytes and plasma cells, was present, sometimes in aggregates (Figure 1C). The presence of collagenic fibrosis was further highlighted by Mallory’s trichrome staining (Figure 1D), and typical fibroblastic foci are displayed in Figure 1E. In some sections, isolated giant multinucleated cells, with cholesterine clefts and poorly formed granuloma, were observed (Figure 1F). Honeycombing or other pathological patterns were absent.

### 3.2. Establishment of a Unique Fibroblastic Cell Line (SCI13D) from fHP Patient

We here report the establishment and characterization of a fibroblastic cell line derived from the broncho-alveolar lavage (BAL) of a patient diagnosed with fHP. The present cell line was continuously expanded until passage 36. Briefly, cells present in BAL were first pelleted by centrifugation and then seeded in a 24-well plate in complete culture medium with the addition of FGF2. After 3 weeks, we could note that a few fibroblasts started to grow and form colonies. These cells, named SCI13D, were continuously expanded in the culture medium with the addition of FGF2 at the suboptimal concentration of 3 ng/mL once a week, in part characterized and frozen. To evaluate proliferation of SCI13D, we first counted the cells to be seeded in a six-well plate at time 0 using trypan blue dye. After 14 days of culture, the expanded cells were detached from each well and collected to determine their number and, therefore, their growth.

**Figure 2:**
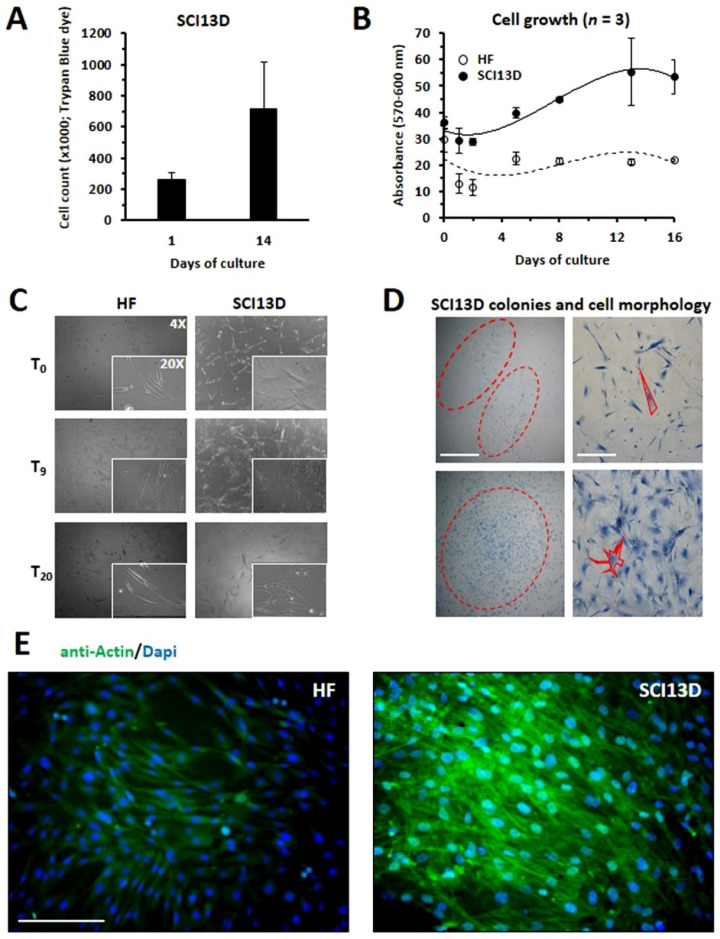
Determination of proliferation and morphological characterization of SCI13D as compared to control fibroblasts. Determination of SCI13D cell line proliferation by (**A**) trypan blue dye exclusion (histograms are the mean of three experiments ± SD), or (**B**) Alamar Blue^TM^ test at different timepoints; (**C**) Morphological representation of growing SCI13D fibroblasts or of control fibroblasts (HF) as observed in bright field; (**D**) SCI13D colony and cell morphology after fixation and staining with Methylene Blue; scale bar = 10 µm; (**E**) actin/DAPI immunofluorescence staining of control fibroblasts (HF) and of SCI13D cell line after their culture in chamber slides; scale bar = 12.5 µm.

**Figure 3:**
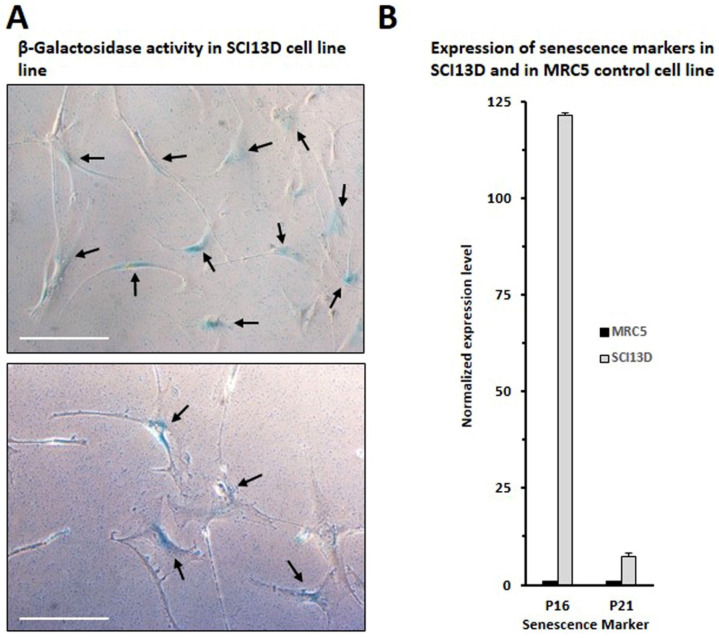
Assessment of senescence markers in SCI13D cell line. (**A**) Evaluation of β-galactosidase activity in SCI13D cell line; scale bar = 25 µm; (**B**) p16 and p21 mRNA levels of expression in SCI13D cell line and in the fibroblast cell line of control MRC5, as detected by quantitative real-time PCR. Histograms depict normalized average values ± SD of three determinations.

As shown in Figure 2A, we observed that the number of SCI13D cells was almost threefold increased at day 14. We also assessed the proliferation of SCI13D using the Alamar Blue^TM^ test, thus comparing, in a time-course experiment, SCI13D growth with that of human skin fibroblasts derived from a healthy donor as control. Figure 2B shows that SCI13D grew moderately faster than the skin fibroblasts used as control (HF). Observations of cultured SCI13D using an inverted light microscope revealed that these fibroblasts gave rise to colonies morphologically more heterogeneous than those formed by HF cells; typical spindle-shaped fibroblasts appeared in fact mixed with a discrete number of larger polygonal cells, which more closely resemble myofibroblasts features (Figure 2C,D). In addition, as shown in Figure 2E, we could note that SCI13D cells tended to overgrow, thus losing contact inhibition, unlike fibroblasts of the control (HF). We further analyzed the expression of β-galactosidase (β-gal), P16, and P21, all of which are considered senescence markers; a consistent number of cells were positive for β-gal (Figure 3A), apparently brighter in enlarged cells with a polygonal morphology. In addition, mRNA levels of the cell-cycle inhibitors P16 and P21 were higher in SCI13D than in the lung control cell line MRC5 (Figure 3B).

**Figure 4:**
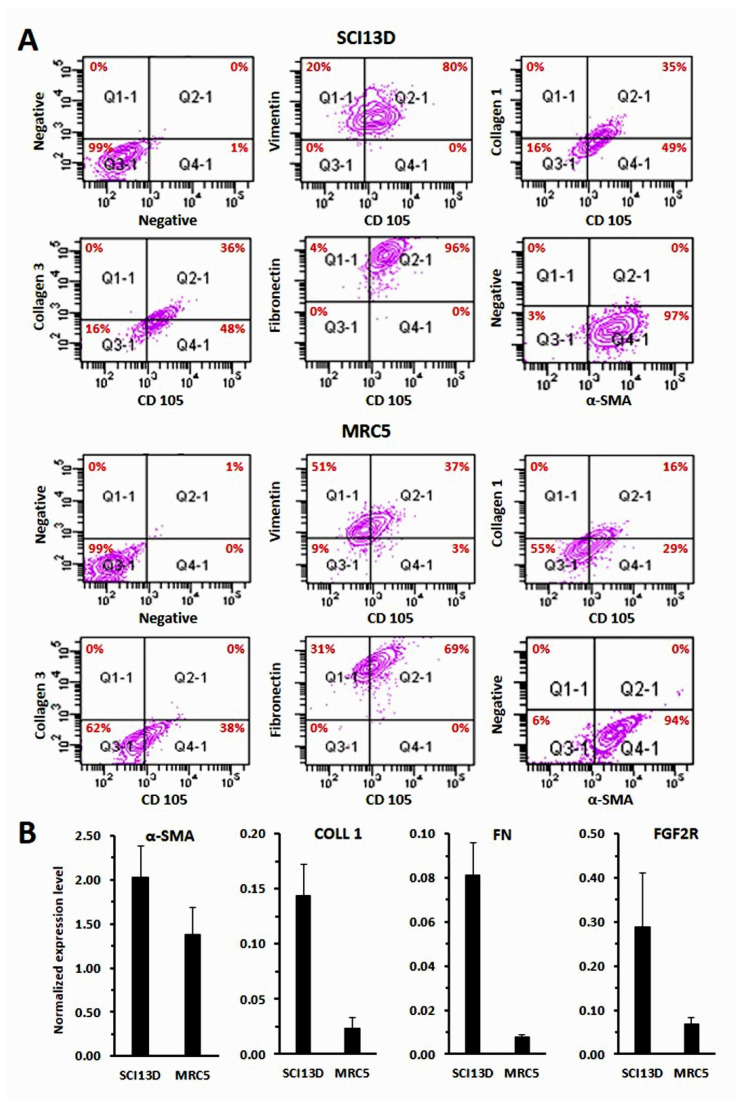
Cyto-fluorographic and RT-PCR determination of typical markers of fibroblasts/myofibroblasts in SCI13D and in MRC5 control cell line. (**A**) Membrane (CD105) and intracytoplasmic detection of vimentin, collagen-1, collagen-3, and α-smooth muscle actin (SMA) in SCI13D as compared with MRC5 cell line, both evaluated at passage 3; (**B**) quantification of mRNA expression levels of α-SMA, type 1 collagen (Coll 1), fibronectin (FN), and FGFR2 in SCI13D with respect to MRC5. Histograms depict normalized average values ± SD of three determinations.

Using cyto-fluorographic analysis (Figure 4A) and quantitative RT-PCR (Figure 4B), we then assessed the expression of markers, such as α-SMA, type 1 collagen, and fibronectin, which are usually upregulated during the differentiation of fibroblasts toward myofibroblasts; all these markers were higher in SCI13D cells than in the MRC5 cell lines, both evaluated at passage 3 (Figure 4A,B). Collagen-1, α-SMA, and fibronectin expression further appeared quite stable along the many passages of the in vitro culture; expression levels of these markers in SCI13D, at early or late passages, presented only a slight increase at passage 30, as compared to passage 4 (Supplementary Figure S1), which is likely to be due to the aging of fibroblasts when cultured in vitro for a long time, as commonly happens in normal control fibroblasts.

We further show here higher mRNA levels of the receptor for FGF2 in SCI13D than in the lung control cell line, which could represent an advantage for cell proliferation. However, it has recently been demonstrated that endogenous overexpression of FGF2 induces myofibroblast dedifferentiation [19]; the role of FGF2 in the fibrotic process of the lung is still unclear and debated [20,21]. Given that we added FGF2 (3 ng/mL) to the culture medium of SCI13D cells, we attempted to clarify whether its presence favored the expansion of dedifferentiated cells. We observed a strong downregulation of the expression of collagen-1 and, to a lesser extent, of α-SMA and fibronectin by FGF2, in basal conditions, as well as after TGFβ stimulation (Supplementary Figure S2). Downregulation of these molecules, as well as the FGF2-induced cell proliferation, detected by Ki67/Coll-1 double staining, was further dose-related (Supplementary Figure S3). The expression of collagen-1 at higher levels was, however, reacquired after culturing SCI13D cells in medium alone for 12 days. (Supplementary Figure S4). These findings collectively highlight the dynamic nature and plasticity of these cells, further indicating that the FGF2-mediated loss of collagen-1 appears as a transient phenomenon. Moreover, the proof that SCI13D cells at the late passage 30 still express collagen-1 at levels as high as at the early passage 4 supports the view that these fibroblasts maintain their features and may effectively be representative of fibroblasts/myofibroblasts classically expanded during the fibrotic process in lung tissue in HP patients.

### 3.3. Immunocytochemical Characteristic of the SCI13D Cell Line

**Figure 5:**
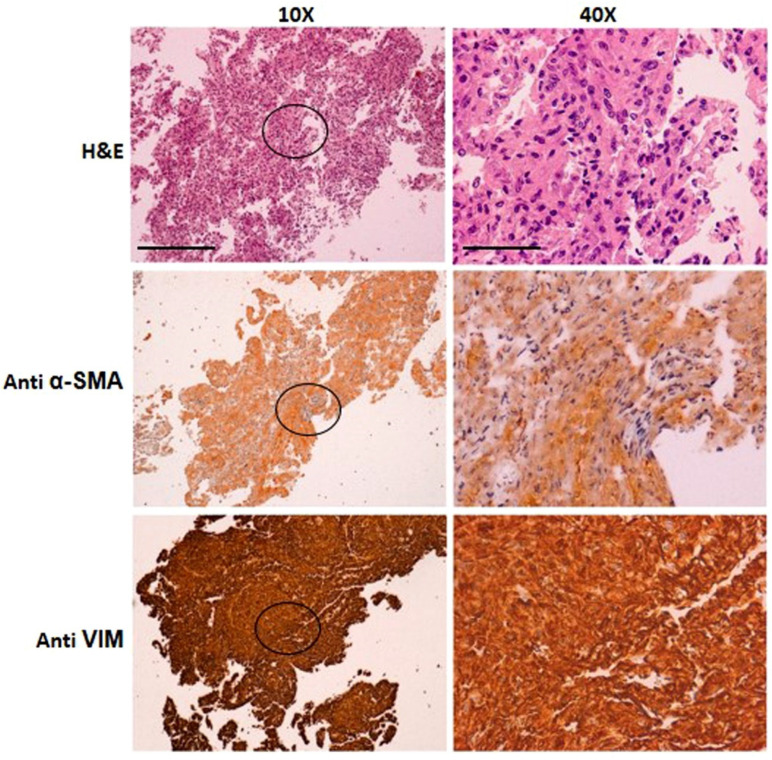
Immunocytochemical characterization of cytocentrifuged samples of SCI13D cells with hematoxylin–eosin (H&E), anti-α-smooth muscle actin (Anti α-SMA), and anti-vimentin (Anti VIM) staining of SCI13D cells; areas indicated in the 10× images (left column; scale bar = 25 µm) are enlarged (40×; scale bar = 6.25 µm) in the right column.

Cytological analysis of the fibroblastic SCI13D cell line evidenced fusiform elements, with elongated nuclei and an eosinophilic cytoplasm. Immunocytochemistry showed strong vimentin positivity and a weaker staining for smooth muscle actin (Figure 5, lower and central rows, respectively), as well as negativity for cytokeratin(s) (*data not shown*). Collectively, these results indicate that the SCI13D line is composed of fibroblasts with partial myofibroblastic differentiation.

### 3.4. Karyotype and FISH

**Figure 6:**
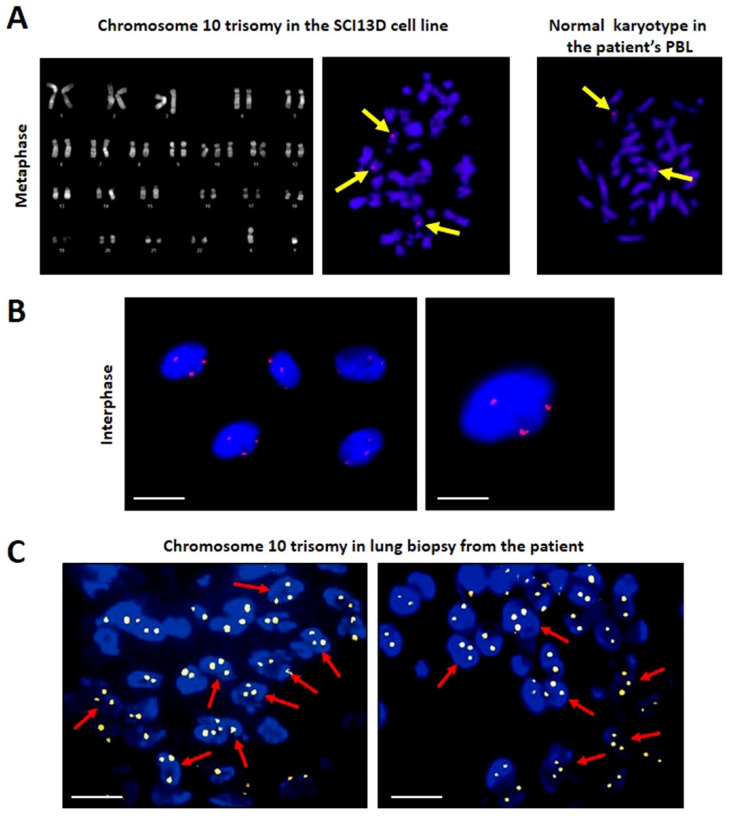
Karyotype of SCI13D cell line. (**A**) Analysis in metaphase of SCI13D cell line identified an aneuploid karyotype with a trisomy of chromosome 10. PBL from the same patient showed instead a normal karyotype in metaphase (100×); (**B**) the presence of three chromosomes 10 was further confirmed by fluorescence in situ hybridization (FISH) of the nuclei of the SCI13D cell line using a specific satellite probe for human chromosome 10; (**C**) chromosome 10 trisomy was already present in vivo in a consistent number of cells in the lung biopsy, as revealed by FISH. (scale bars in B, left panel, and C = 5 µm; scale bar in B, right panel, =2.5 µm).

In SCI13D, we detected an aneuploid karyotype with a trisomy of chromosome 10 (46XY + 10) by G banding karyotype analysis (Figure 6A, left panel). This finding was then confirmed by fluorescence in situ hybridization (FISH) performed with a specific probe detecting chromosome 10, in metaphase (Figure 6A, central and right panels) or in interphase (Figure 6B). A normal karyotype was instead found in PHA-activated peripheral blood lymphocytes (PBLs) obtained from the same patient (Figure 6A, right panel). Trisomy of chromosome 10 was already observable at the early passages of in vitro expansion (passage 3), intriguingly suggesting that this anomaly was not acquired during their long-term culture. This suggestion was also supported by the detection of fibroblasts with trisomy 10 in vivo, within the patient’s biopsy, as shown in Figure 6C. It is worth noting that the trisomy of chromosome 10 was observed in nearly 99% of the nuclei analyzed, and that this percentage was stable along the many passages in culture, thus indicating that these fibroblasts appear representative of a clonal population and exhibit stable features.

### 3.5. Authenticity of the SCI13D Cell Line

The authenticity of the SCI13D cell line was verified through short tandem repeat (STR) profiling by the Banca Cellule Interlab Cell Line Collection (ICLC; IRCCS Ospedale Policlinico San Martino, Genoa, Italy), thus providing the true origin and a unique profile of these cells, with respect to the profiles present in the reference databases (Table 1).

### 3.6. Inhibition of Proliferation by Antifibrotic Therapy

**Figure 7:**
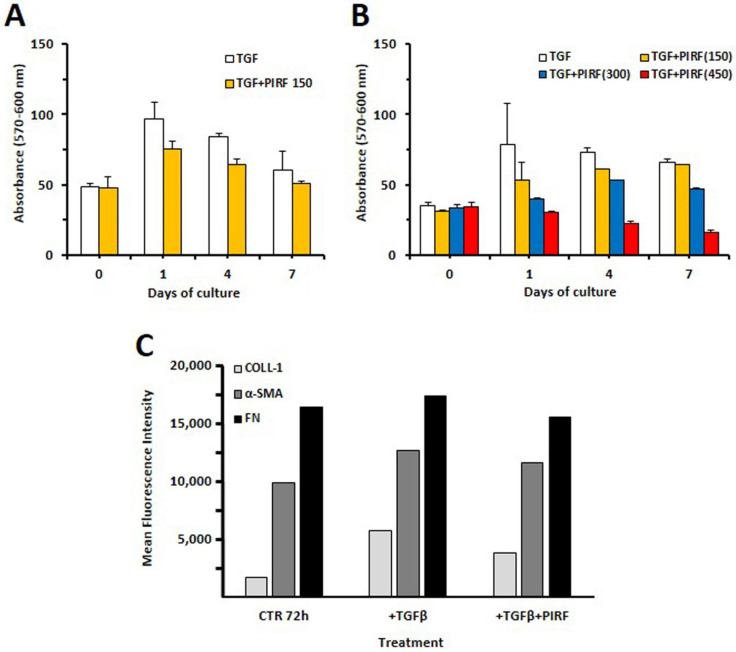
Evaluation of the TGF-β-induced proliferation or of the expression of typical myofibroblast markers in SCI13D in the presence/absence of pirfenidone treatment. (**A**) Pirfenidone (150 μg/mL; PIRF) consistently reduced TGF-β-stimulated SCI13D proliferation as evaluated by Alamar Blue^TM^ test at different timepoints; (**B**) inhibition of SCI13D cell proliferation resulted further strongly enhanced when pirfenidone was used at a higher concentration (i.e., 450 μg/mL completely blocked cell growth). Histograms depict mean values of triplicate analysis; (**C**) pirfenidone strongly diminished TGF β-induced expression of type-1 collagen and, to a lesser extent, that of α- SMA or FN in SCI13D cells.

Trying to explore whether the potential use of pirfenidone could be of help in limiting pulmonary fibrosis in fHP patients, we tested this drug in TGF β-stimulated SCI13D cells. As shown in Figure 7A,B, pirfenidone exerted a consistent inhibition of SCI13D cell growth, which was dependent on the concentration of the drug added at the beginning of the cell culture (150–450 μg/mL). The treatment with 450 μg/mL completely blocked cell proliferation along 7 days of culture, while the growth of fibroblasts exposed to 150–300 μg/mL was only partially inhibited.

### 3.7. Pirfenidone Slightly Inhibited Type 1 Collagen Expression in TGF-β-Treated SCI13D Cells

We further evaluated if pirfenidone could modulate the expression of type 1 collagen and fibronectin. We detected upregulation of type 1 collagen, α- SMA, and FN expression with TGF β (5 ng/mL) in SCI13D. The contemporary administration of pirfenidone (150 μg/mL) caused a more remarkable downregulation of collagen-1 than α- SMA or FN (Figure 7C).

### 3.8. Inhibition of SCI13D Migration by Pirfenidone

**Figure 8:**
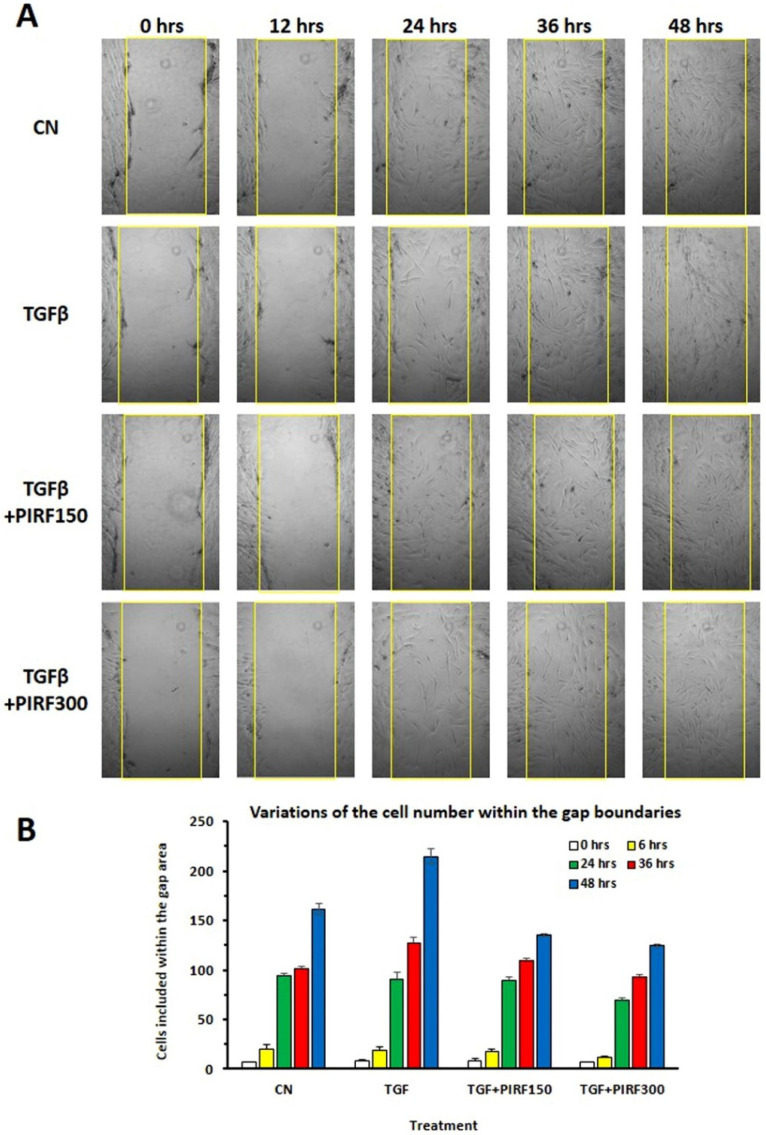
Wound scratch assay. (**A**) The migratory capacity of the SCI13D cells, in the presence or absence of TGF-β and/or pirfenidone (PIRF), was assessed by acquiring images of the cells filling in the lesion gap (yellow lines) at the indicated timepoints (0, 6, 24, 36, and 48 h); (**B**) histograms quantify the cell numbers within the lesion boundaries, at each timepoint, for each treatment. Cells were scored in three images for each timepoint and treatment.

We studied the effect of pirfenidone on SCI13D migration by utilizing a wound scratch assay and then measuring the cell migration rate toward the injured site. Pirfenidone decreased basal and TGF-β-induced migration, and the inhibition was particularly evident after 48 h at the concentration of 300 μg/mL (Figure 8).

### 3.9. Reduction of the TGFβ-Induced Profibrotic Phenotype by Pirfenidone in SCI13D Cell Line

**Figure 9:**
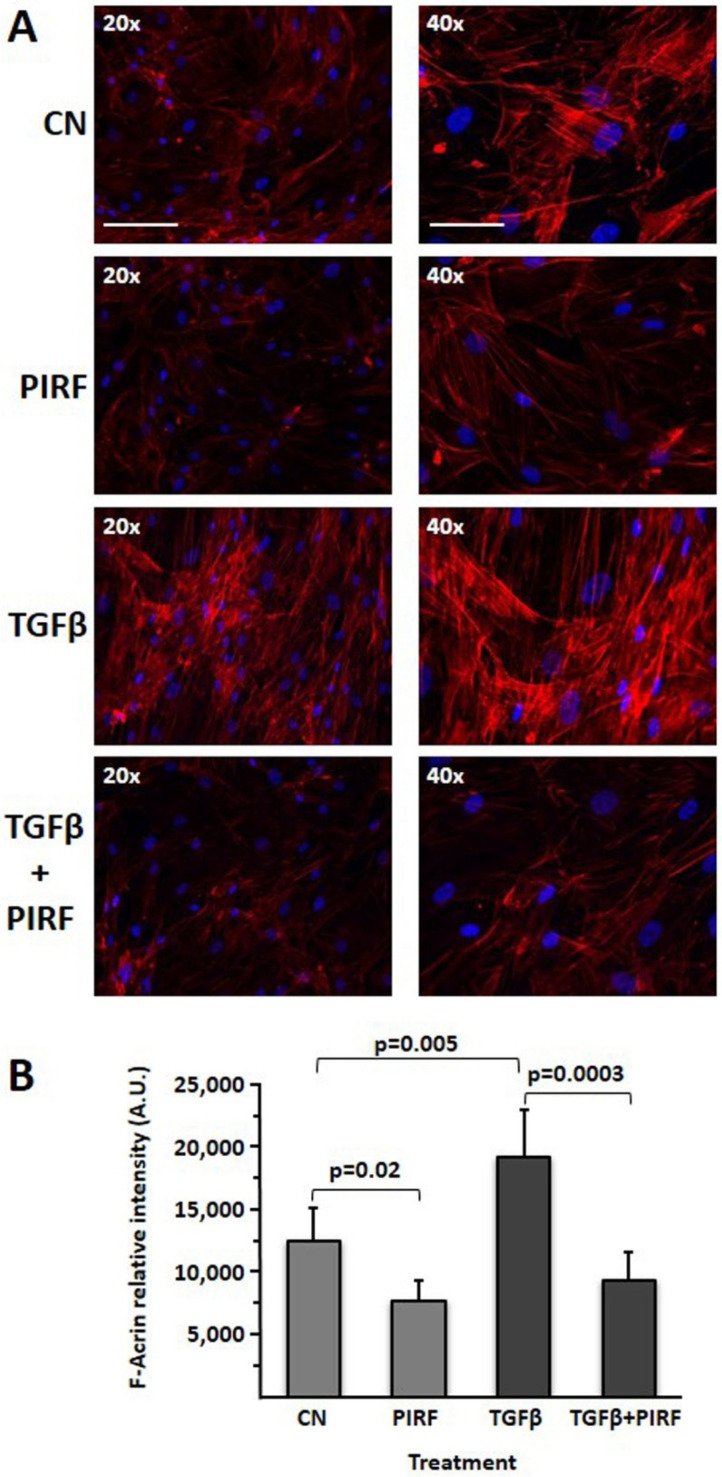
Evaluation of the expression of F-actin in SCI13D cells treated or untreated with TGFβ or TGFβ + Pirfenidone (**A**) Pirfenidone (PIRF) consistently reduced F-actin fiber fluorescence in SCI13D fibroblasts treated at basal conditions as well as stimulated with TGFβ. (**B**) Determination of F-actin relative intensity. Histograms are the average ± SD of six different images acquired in duplicate wells for each treatment after 72 h.; scale bars = 12.5 µm and 6.25 µm for the left and right columns, respectively.

We then assessed the effect of pirfenidone in SCI13D cytoskeleton reorganization. The profibrotic myofibroblast phenotype, strongly evident in TGF-β-stimulated cells, was significantly reduced after pirfenidone treatment, thus showing a consistent inhibition of F-actin stress fiber formation. A discrete inhibition of F-actin fluorescence was also observable in cells cultured with pirfenidone in basal conditions (Figure 9A,B). These findings altogether suggest that pirfenidone may limit differentiation toward myofibroblasts, possibly reverting their typical features through contractile structure disassembly.

## 4. Discussion

A complex interplay of genetic, host, and environmental factors influences the development of HP, and the mechanisms that trigger irreversible fibrotic progression in this disease are still uncertain. During chronic inflammation, fibroblasts expand to differentiate into myofibroblasts, thus producing a large amount of extracellular matrix and finally leading to the destruction of the lung tissue. In fibrotic hypersensitivity pneumonitis, the source of fibroblasts is still unclear. They may possibly derive from local or migrated mesenchymal cells, from bone-marrow fibrocytes, attracted in loco by an imbalanced production of chemokines, or from epithelial cells differentiated into mesenchymal cells (epithelial–mesenchymal transition; EMT). Here, we report the establishment and characterization of a fibroblastic cell line (SCI13D) derived from the broncho-alveolar lavage of a patient with fHP. This cell line displays features of myofibroblasts; we in fact detected higher mRNA levels of α-SMA, as well as of type 1 collagen and fibronectin, than in the control cell line MRC5. From a morphological point of view, we observed spindle-shaped cells mixed with enlarged flattened cells, which could more likely resemble the myofibroblast morphology. A consistent number of SCI13D cells show β-galactosidase activity, and mRNA levels of the two cell-cycle inhibitors P16 and P21, typical markers of senescence, were also higher than in the MRC5 cell line used as control. While a close link between senescence and IPF has been previously well established [22,23], robust evidence supporting an association between cellular senescence and progressive fibrosis in fHP is still missing. Interestingly, fibroblasts and epithelial cells within fibroblastic foci observed in lung samples from IPF patients were P16-positive. In addition, the proof that the secretome of senescent fibroblasts is profibrotic appears of particular relevance [24]. The findings of abnormal shortening of telomeres, observed in a number of fHP patients [25,26], as well as the demonstrations that the response to inhalational antigens differs in young and in old mice or that fibrosing HP is mostly diagnosed in elder patients [27,28,29] might indeed suggest a relationship between cellular senescence and fibrosis. Further studies will be of help to deeply clarify whether and how senescent cells might contribute to progressive fibrosis in HP or to determine if the usage of senolytic drugs, clearing senescent cells, could attenuate lung injury and inhibit fibrosis.

In SCI13D, the early appearance of an aneuploid karyotype is also intriguing; proof of the presence of a trisomy 10 within the lung biopsy demonstrated that this anomaly is already present in vivo as a somatic mutation, possibly characterizing at least a part of the patient’s fibroblasts. We still do not know whether this anomaly provides a selective advantage to cell expansion in vivo or in vitro. Given that chromosome 10 harbors the FGF2R gene, presumably higher levels of this receptor, due to the presence of an extra chromosome 10, may lead to overgrowth of a proliferative clone. Although it is still unclear how aneuploidies may control the expression and transcriptional activities of the genes encompassed within the extra chromosome, Sunyoung Hwang and coworkers recently demonstrated that their mRNA levels increase proportionally with gene copy numbers and that mechanisms required to compensate for the gene expression of an extra copy of a human autosome do not seem to be engaged [30]. SCI13D expressed higher mRNA levels of FGFR2 than the MRC5 lung control cell line; however, further investigations would be of help to better establish a potential link between the presence of an extra chromosome 10 and its putative selective advantage for cell expansion.

FGF2 has been reported to be profibrotic [31], but it was also recently demonstrated that its overexpression may instead protect from bleomycine-induced pulmonary fibrosis [19], through promotion of proliferation and myofibroblast dedifferentiation [32]. FGF2 may in fact lead to downregulation of collagen-1, α-SMA, and fibronectin in myofibroblasts, thus reverting their phenotype and features. We report here that the addition of FGF2 in culture affects the levels of expression of these markers in the SCI13D cell line, especially for collagen-1, but only in a transitory manner. This finding, together with the proof that SCI13D at late passages does still express at high levels the typical myofibroblast markers, allows excluding the selection of dedifferentiated cells along the in vitro culturing process. It is, however, conceivable that FGF2 might promote a small fraction of cells as a less differentiated proliferating reservoir, as already demonstrated for the expansion of mesenchymal cells [33]. Hence, in this pathology, the specific role of FGF2 in fibrosis development in promoting myofibroblast dedifferentiation to resolve fibrosis deserves further attention. It is, however, likely that, in vivo, an imbalance in concentrations of cytokines, present in the microenvironment, particularly TGFβ and FGF2, may influence the course of pulmonary fibrosis, as well as the response to therapy.

The antifibrotic drug nintedanib was recently shown to be effective at slowing disease worsening in individuals with progressive fibrosis due to any cause [34]. Similarly, pirfenidone was demonstrated to improve lung function decline in patients with unclassifiable ILD [35], but further studies will better clarify which treatments should be considered the first line in fHP and whether immunosuppressants should be combined with antifibrotic drugs [36]. Although pirfenidone is deemed to be effective in IPF and the compound is now in widespread clinical use, to date, its molecular activities are only partly defined; apposite investigations are required to purpose pirfenidone for the treatment of other respiratory diseases. Indeed, pirfenidone was recently proposed as an implemental treatment to disrupt tumor–stroma interactions in non-small-cell lung cancer [37], and novel in vitro models might, therefore, be of help to better define its utility. The effectiveness of pirfenidone in inhibiting SCI13D proliferation was demonstrated here as a dose-dependent inhibition, especially when 450 μg/mL pirfenidone was added at the beginning of the culture. We also observed that pirfenidone downregulated the TGF-β-induced expression of collagen-1, inhibited migration of SCI13D in a wound scratch test, and induced cytoskeleton re-organization, thereby reducing F-actin stress fiber expression.

In-depth characterization of fHP fibroblasts might be limited by the short life of these cells when cultured ex vivo. The availability of a great number of cells may, therefore, allow to better characterize fHP fibroblasts, to define molecular mechanisms regulating their interactions with epithelial and/or endothelial cells in 2D/3D models, or to devise lung-tissue-like organoids. Collectively, these in vitro models might be helpful for drug screening and preclinical studies. In this context, the SCI13D cell line, displaying features comparable to primary cells but with improved proliferation capacity, may represent a suitable tool.

## 5. Conclusions

In conclusion, we believe that the cell line here reported may provide a useful tool to study mechanisms of progressive fibrosis in HP disease. In vitro models, mimicking the in vivo situation, could be of help to study disease-relevant cell–cell interactions and to explore novel therapies. Therefore, the SCI13D cell line might be valuable in the generation of 2D or even more complex 3D models of lung-like tissue.

## Figures and Tables

**Table 1:** STR profile of the assessed cell lines. Fifteen highly polymorphic STR loci plus amelogenin (AM) were used.

	Cells Assessed	
STR Locus	PBL	SCI13D
D5S818	11,12	11,12
D13S17	9,11	9,11
D7S820	11	11
D16S539	9,14	9,14
VWA	16	16
TH01	6	6
AM	x, y	x, y
TPOX	9,10	9,10
CSF1PO	11,12	11,12
D21S11	27,30	27,30
D3S1358	16	16
D18S51	13,16	13,16
Penta E	7,17	7,17
Penta D	9,11	9,11
D8S1179	14,15	14,15
FGA	19,22	19,22

## Supplementary Materials

The following are available online at https://www.mdpi.com/article/10.3390/biomedicines9091193/s1, Figure S1: Comparison of myofibroblast markers between early and late passages of SCI13D cell line; Figure S2: Evaluation of defects of FGF-2 stimulation on the modulation of myofibroblasts (Col 1, α-SMA, FN) and senescence (p21) markers in SCI13D at different passages (p7 and p31, panel A and B respectively); Figure S3: Determination of FGF2-stimulated proliferation by Trypan Blue cell counts and by Ki-67 staining and cytofluorimetric analysis; Figure S4: Recovery of the expression of Collagen 1 after 12 days from the FGF2 stimulation of SCI13D p4.
