# Supplementary material for: Establishment and Characterization of a Novel Fibroblastic Cell Line (SCI13D) Derived from the Broncho-Alveolar Lavage of a Patient with Fibrotic Hypersensitivity Pneumonitis
Source: Biomedicines. 2021 Sep 10;9(9):1193. doi: 10.3390/biomedicines9091193 (PMC8465388; doi:10.3390/biomedicines9091193)
Supplement: Supplementary file 1 [file biomedicines-09-01193-s001.zip › biomedicines-1336786-supplementary.pdf]

Supplementary material

Establishment and characterization of a novel fibroblastic cell line (SCI13D) derived from the broncho-alveolar lavage of a patient with fibrotic Hypersensitivity Pneumonitis

Paolo Giannoni [1,£], Marco Grosso [2,£], Giuseppina Fugazza[3], Mario Nizzari [4], Maria Cristina Capra [5], Rita Bianchi [6], Roberto Fiocca [6,7], Sandra Salvi [6], Fabrizio Montecucco [8,3], Maria Bertolotto [3], Franco Fais [1,5], Mario Salio [9], Emanuela Barisione [2, §], Daniela de Totero [5, §,*]

Supplementary Figure 1

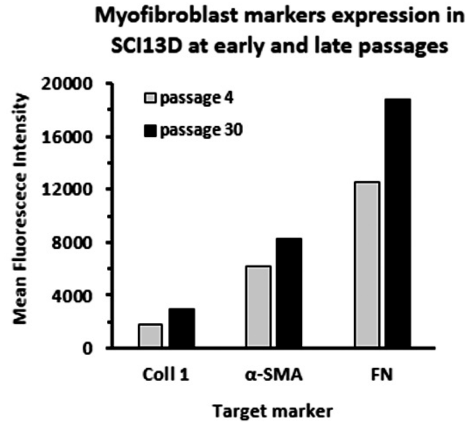

Supplementary Figure S1

Comparison of myofibroblast markers between early and late passage of SCI13D cell line. The expression of typical markers of myofibroblasts (Collagen 1 (Coll 1), α-SMA and Fibronectin (FN)) was evaluated in SCI13D at early (passage 4) or late (passage 30) passages by means of cytofluorimetric analysis.

Supplementary Figure 2

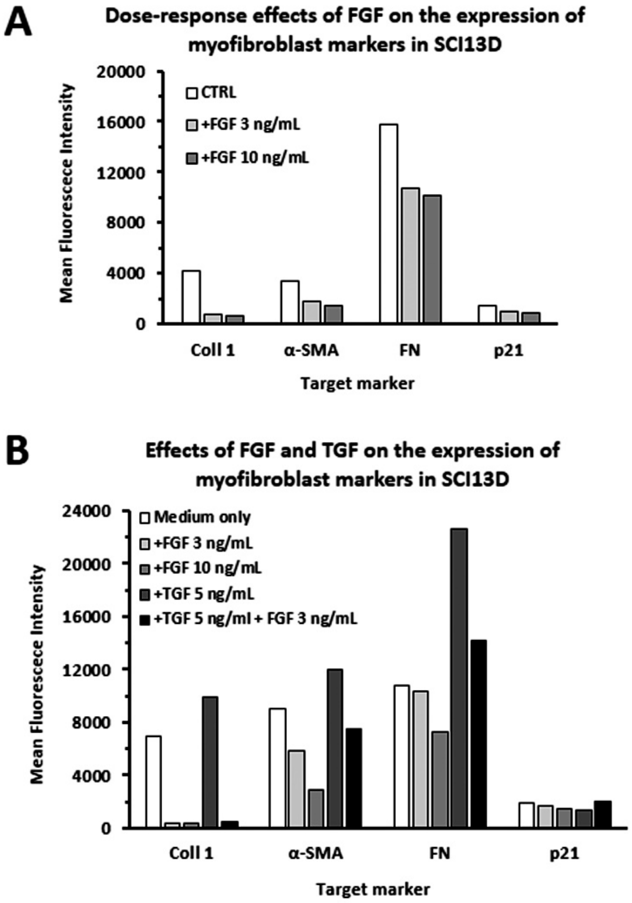

Supplementary Figure S2

Evaluation of the effects of FGF2 stimulation on the modulation of myofibroblasts (Coll 1, α-SMA, FN) and senescence (p21) markers in SCI13D at different passages (p7 and p31, panel A and B respectively). A: Stimulation of FGF2 at 3 or 10 ng/ml for 72h strongly down-modulated Collagen-1 and to a lesser extent α-SMA and Fibronectin. P21 was almost unchanged. **B**: FGF2 consistently downregulated Collagen-1 at basal conditions as well as after TGFβ treatment. P21 appeared stably expressed independently of any treatment. During the first 4 days of culture the cells were exposed to culture medium only and then subjected for 72h to the various experimental conditions as depicted in the figure.

Supplementary Figure 3

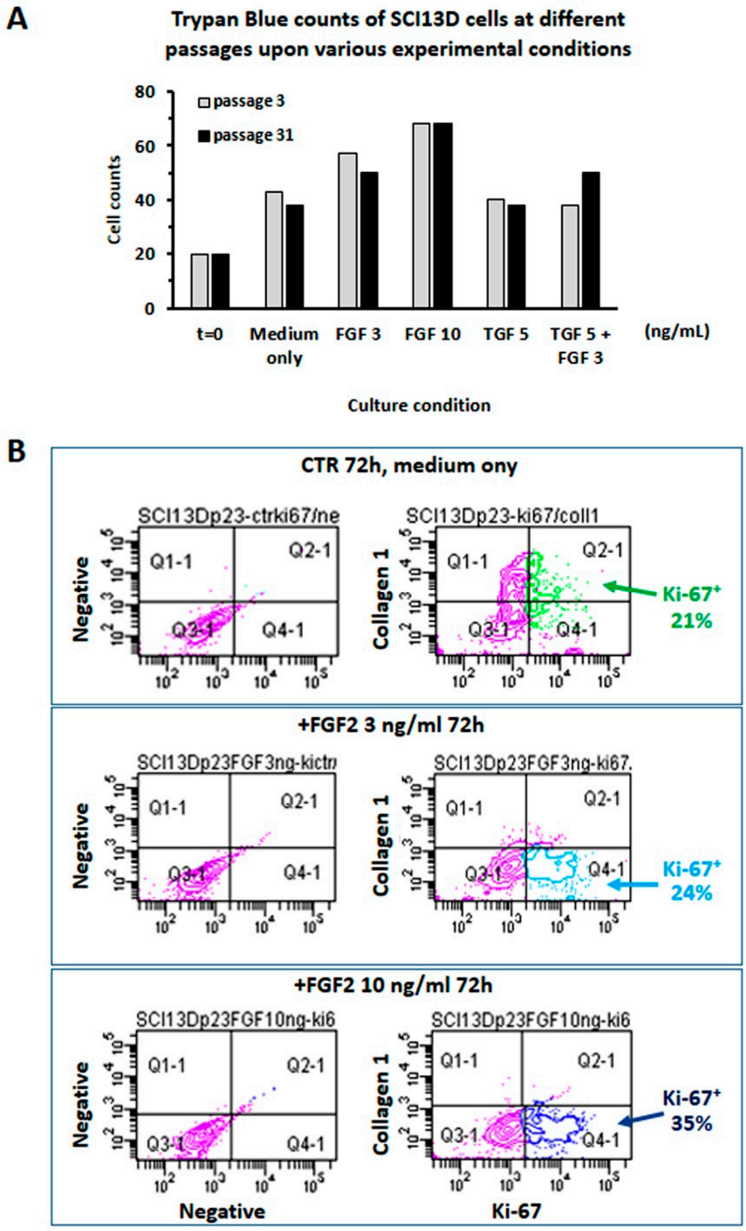

Supplementary Figure S3

Determination of FGF2-stimulated proliferation by Trypan blue cell counts and by Ki-67 staining and cytofluorimetric analysis. A: The index of proliferation of SCI13D at different passages (p3, p21) plated at 20000 cells/ml at time 0 was determined by Trypan blue dye after 7 days of culture. During the first 4 days the cells were exposed to culture medium only and then subjected for 72h to various experimental conditions (medium culture or FGF2 at 3 or 10 ng/ml, TGFβ at 5 ng/ml or FGF2+TGFβ at 3 and 5 ng/ml respectively). **B**: Ki67/Collagen-1 double staining of SCI13D (p23) at basal condition (CTR 72h) or after 72h-culture with FGF2 (3 or 10 ng/ml). The percentage of double stained cells are indicated and shows a FGF2-dose-dipendent increase in the proliferating cell fraction.

Supplementary Figure 4

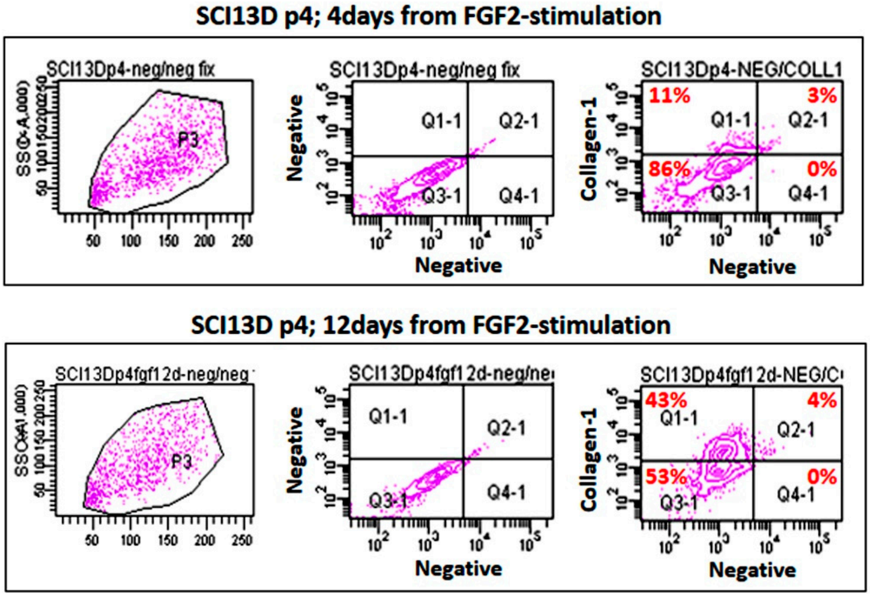

Supplementary Figure S4

Recovery of the expression of Collagen-1 after 12 days from the FGF2 stimulation of SCI13D p4. After 12 days the percentage of cells expressing Collagen 1 was 43% (lower panel) as compared to 11% only after 4 days from FGF exposure (upper panel).
